# Rearrangement in the Hypervariable Region of JC Polyomavirus Genomes Isolated from Patient Samples and Impact on Transcription Factor-Binding Sites and Disease Outcomes

**DOI:** 10.3390/ijms23105699

**Published:** 2022-05-20

**Authors:** Michael P. Wilczek, Aiden M. C. Pike, Sophie E. Craig, Melissa S. Maginnis, Benjamin L. King

**Affiliations:** 1 Department of Molecular and Biomedical Sciences, University of Maine, Orono, ME 04469, USA; michael.wilczek@maine.edu (M.P.W.); aidan.pike@maine.edu (A.M.C.P.); sophie.craig@maine.edu (S.E.C.); 2 Graduate School in Biomedical Science and Engineering, University of Maine, Orono, ME 04469, USA

**Keywords:** JC polyomavirus, transcription factors, mutations, viral genome, PML, NCCR

## Abstract

JC polyomavirus (JCPyV) is the causative agent of the fatal, incurable, neurological disease, progressive multifocal leukoencephalopathy (PML). The virus is present in most of the adult population as a persistent, asymptotic infection in the kidneys. During immunosuppression, JCPyV reactivates and invades the central nervous system. A main predictor of disease outcome is determined by mutations within the hypervariable region of the viral genome. In patients with PML, JCPyV undergoes genetic rearrangements in the noncoding control region (NCCR). The outcome of these rearrangements influences transcription factor binding to the NCCR, orchestrating viral gene transcription. This study examines 989 NCCR sequences from patient isolates deposited in GenBank to determine the frequency of mutations based on patient isolation site and disease status. The transcription factor binding sites (TFBS) were also analyzed to understand how these rearrangements could influence viral transcription. It was determined that the number of TFBS was significantly higher in PML samples compared to non-PML samples. Additionally, TFBS that could promote JCPyV infection were more prevalent in samples isolated from the cerebrospinal fluid compared to other locations. Collectively, this research describes the extent of mutations in the NCCR that alter TFBS and how they correlate with disease outcome.

## 1. Introduction

JC polyomavirus (JCPyV) is a human-specific virus that infects most of the population [1,2,3]. JCPyV was first identified upon isolation from the brain of a patient with Hodgkin’s disease that had developed progressive multifocal leukoencephalopathy (PML) [4]. Since this discovery, JCPyV has been found to cause a persistent infection in the kidneys of healthy individuals, and, during immunosuppression, reactivate and spread in the central nervous system (CNS) causing the fatal, demyelinating disease, PML [1,5,6,7,8]. There are currently no approved cures for PML, and when left untreated, the disease can be fatal within a few months [9,10]. Historically, individuals most at risk for disease were positive for HIV, representing up to 5% of all PML cases [3,9]. The use of highly active antiretroviral therapy (HAART) has significantly reduced the rate of PML among HIV individuals [11,12]. Unfortunately, new risk groups are emerging that encompass patients receiving immunomodulatory therapies for immune-mediated diseases [13]. This includes individuals with multiple sclerosis (MS) taking natalizumab and individuals receiving rituximab for treatment of systemic lupus erythematosus (SLE) [9,13,14,15]. As there are no approved therapies for PML, current medical interventions address treatment of the underlying immunosuppression by either treating HIV with HAART or discontinuing immunosuppressive therapies [9,10,16,17]. Recently, there have been treatments related to adoptive T cell transfer, and checkpoint inhibitors, such as pembrolizumab, however these treatments can also result in severe morbidity [9,18,19,20].

The pathogenicity of JCPyV, infecting most of the population and causing disease in immunosuppressed hosts, is determined not only by the underlying health of the host but also by the viral genome. JCPyV is a small, nonenveloped double-stranded DNA virus with a circular genome of approximately 5100 bp in size [21,22]. Transcription and replication occur in the nucleus and are orchestrated by the noncoding control region (NCCR) [8,23]. The NCCR divides the viral genome into early and late regions, with the early genes serving to transform the cell into a supportive environment and regulate downstream steps in the replication cycle, and late genes driving subsequent stages of infection, including viral assembly and release [23].

Within the nucleus, host transcription factors (TFs) bind to the NCCR to initiate transcription of the early genes, Large T Antigen (T Ag), small t antigen (t ag), and three alternatively spliced transcripts, T’135, T’136, and T’165 [24]. T Ag is a multifunctional protein that is critical in establishing a conducive environment for viral replication. First, it binds to retinoblastoma (pRB), causing the release of the transcription factor, E2F-1, and inducing the cell into S phase [25,26]. Secondly, T Ag can also bind to p53, blocking the cell from activating apoptotic pathways, and lastly, it can act as a helicase, unwinding the viral DNA to continue the production of virus progeny [27,28]. t ag and the spliced variants facilitate T Ag in driving the cell cycle into S phase and mediate interactions with p107 and p130, related to the Rb family [29,30]. Once viral DNA is replicated in the nucleus, the late viral genes are transcribed, producing the structural proteins: viral protein 1 (VP1), VP2, and VP3, as well as a multifunctional protein, agnoprotein [23]. VP1 is the major component of the capsid; the viral capsid consists of 72 VP1 pentamers each interacting with a minor capsid protein, VP2 or VP3 [31]. Like T Ag, agnoprotein is also considered to be a multifunctional protein. Research has demonstrated that agnoprotein can interact with T Ag, suppressing viral DNA replication and help transition the cell into G2/M phase, allowing the viral DNA to be encapsidated by the VPs [32]. Additionally, agnoprotein can act as a viroporin, facilitating viral release from the cell [33].

DNA viruses, including JCPyV, are completely dependent on host TFs to initiate and coordinate the viral infectious cycle. Host TFs bind to the NCCR within the JCPyV genome, and this small region of the viral genome that is only ~145 base pairs, but can vary, is not only highly important in the infectious cycle but is a strong indicator of viral disease [23,34]. Previous research has demonstrated that 100% of JCPyV isolates from patients that were diagnosed with PML had genetic mutations and rearrangements in the NCCR compared to non-disease isolates and to other regions of the viral genome, such as VP1 [34,35]. The NCCR is characterized as having an early and late proximal region. The early region contains the origin of replication (ORI), binding sites for transcription of early genes, and binding sites for T Ag to help regulate viral infection [24]. Due to these functions all having important roles in establishing infection, the early proximal region is highly conserved among polyomaviruses and is not prone to mutation [36]. However, this is in considerable contrast to the late proximal region. The late region is hypervariable, undergoing mutations and rearrangements that can account for increased viral gene expression and enhanced tissue tropism and disease pathogenesis [24,37,38]. The JCPyV NCCR is divided into DNA sequence blocks denoted as lowercase letters “a”, “b”, “c”, “d”, “e”, and “f”, which can undergo rearrangements [39,40,41]. The non-pathogenic strain, also called the archetype or CY, is found predominately in the kidneys (or shed in the urine) of healthy individuals and has all six DNA sequence blocks in alphabetical order [13,42]. The viral sequences of isolates associated with PML, also known as the PML-type, have significantly rearranged NCCRs [24]. The prototype JCPyV NCCR sequence is designated by the Mad-1 variant, that was first isolated from a PML patient [4,23]. Mad-1 has deletions in the “d” block and is composed of only blocks, “a”, “c”, and “e” as 98-bp tandem repeats followed by block “f” [13,24,43]. There are numerous JCPyV isolates, including other Mad-isolates that were derived from tissues of PML patients [44], with Mad-8, having similar blocks to Mad-1 but also having a portion of block “b” as well as insertions of single base pairs; Mad-8 is more typical of NCCR variants found in PML patients, compared to Mad-1 [13,44,45]. Additionally, beyond the consensus sequences of JCPyV isolates, deep sequencing analysis of the NCCR has demonstrated that JCPyV isolated from urine closely resembles the CY strain, while isolates from the CSF and plasma of PML patients have significantly rearranged NCCRs with a highly diverse viral population representing a quasispecies [42]. 

Pathogenic isolates share a 98-base pair direct tandem repeat, referred to as an enhancer element in the NCCR. These enhancer elements are composed of blocks, “a”, “c”, and “e” and therefore, contain duplicate TATA boxes, located in the “a” block, and have additional transcription factor binding sites (TFBS) [37,46,47]. Due to the prevalence of these tandem repeats in the NCCR sequences of viral isolates from PML patients, as well as the archetype rarely associated with PML tissue [13,24,47], it is suggestive that these enhancer elements and the addition of TFBS are critical for viral pathogenesis. Furthermore, the loss of the 23-base pair “b” block and the 66-base pair “d” block can also result in increased viral gene expression. These deletions of both regions in the NCCR allow for additional TFs such as YB-1/Purα and NF-1, to facilitate enhanced viral gene expression [38,48]. TFs, such as Spi-B transcription factor (Spi-B), nuclear factor of activated T cells 4 (NFAT4) and subtypes of the nuclear factor 1 (NF-1) family are also important in early gene transcription and may also play a role in cellular tropism [49,50,51,52]. Specifically, JCPyV can infect B cells, and as these cells mature, TFs such as NFI-X and Spi-B have been shown to be upregulated [51,53]. These changes can enhance viral transcription and most notably in individuals who are receiving natalizumab treatment, induce B cell differentiation, possibly inducing the development of PML [54].

Associations between the genetic mutations in the NCCR and PML have been described previously, and there is comprehensive evidence to highlight TFs that are important for JCPyV infection. However, metanalyses to observe the changes in the NCCR in the archetype and PML-type strains as well as tissue location of sequence isolates are very limited. A recent study by Nakamichi et al. described the curation of a database with TFBSs in NCCR sequences of archetype and PML-type JCPyV isolates identified through computer simulations [55]. Our current study provides an extensive bioinformatic approach to validate and uncover TFBS that are influenced by NCCR rearrangements which ultimately enhance viral transcription and cause disease. Using published sequences isolated from deidentified patient samples, this study characterizes the NCCRs of 989 nucleotide sequences, defining them by both their location of isolation and disease status. Most importantly, by using the largest open-access database of curated and non-redundant TFBS, known as JASPAR [56], this study validated and elucidated possible novel TFBS that influence JCPyV infection in each of the six blocks that arise from rearrangements in the NCCR. These data will provide additional rationale and framework to understand how this hypervariable region of the JCPyV genome can persist in almost 80% of the population, however mutations in the NCCR can enhance viral infection, expand tissue tropism, and ultimately, cause the fatal disease PML. 

## 2. Results

### 2.1. The Curation of 989 JCPyV NCCR Sequences from Patient Samples 

A total of 989 unique NCCR sequences were identified from 5507 JCPyV sequences downloaded from GenBank on 6 December 2021. Of the 989, 579 were GenBank records containing only the NCCR region. In total, 410 additional NCCR sequences were extracted from GenBank records that contained JCPyV whole genome assemblies. The NCCR was taken as the interval from the origin of replication to the coding sequence of the agnoprotein. Tissue source and disease status was determined from the GenBank record (Table 1 and Supplemental Table S1) [12,23]. There were 565 nonurine sequences, 217 from the cerebral spinal fluid (CSF), 111 from the plasma, serum, or peripheral blood mononuclear cells (PBMCs), and 32 sequences from the brain. A total of 45.6% (451/989) patients had PML regardless of isolate site, and we also noted patients with secondary diseases where possible (Table 1). One hundred and seventy-nine sequences isolated from urine were from healthy patients and the remaining sequences were further divided by disease status as the archetype strain can be detected in the urine [57]. Collectively, using sequences readily available in GenBank, we curated a set of 989 NCCR sequences isolated from urine, CSF, brain, and plasma, from both healthy and diseased patients (Supplemental Table S1).

### 2.2. NCCR Sequences from PML Patients Derived from Urine Samples Were Distinct from Those from CSF, Brain, and Plasma Samples 

**Figure 1:**
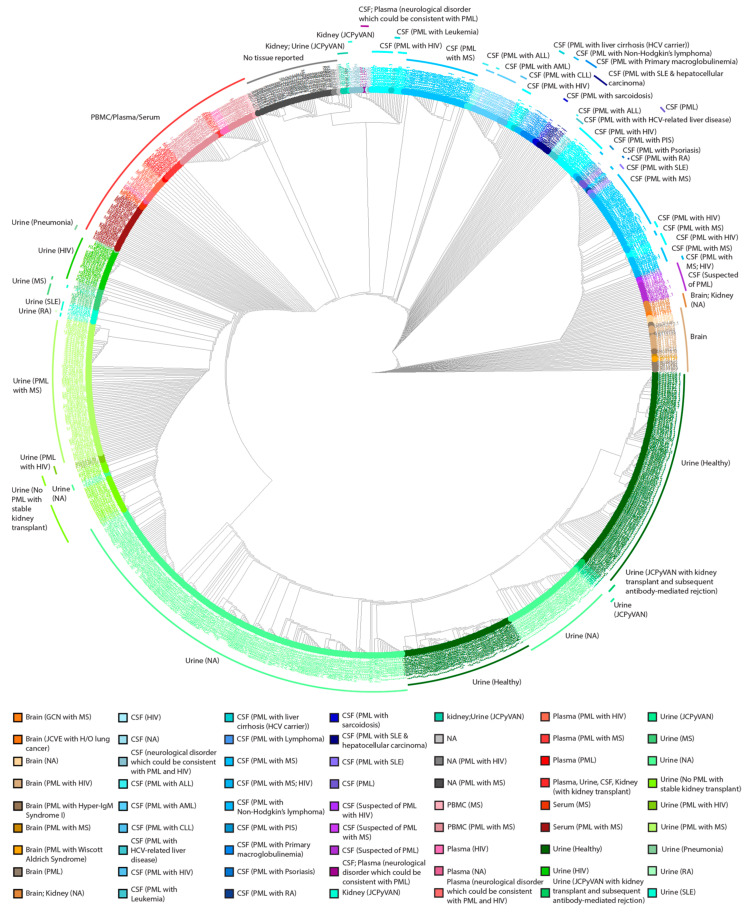
Cladogram of 989 NCCR sequences labeled with the sample isolation site and the disease status of the patients. 989 JCPyV NCCR sequences were aligned using Clustal Omega using the EMBOSS package. Colors used in the circular cladogram created using the R/ggtree package correspond to the site of sample isolation and disease status of individual patients.

A cladogram of the 989 NCCR sequences was performed to cluster the sequences based on similarity. The sequences were aligned with Clustal Omega [58] and the neighbor joining algorithm was used to generate a cladogram that was then labeled by sample site and disease status (Figure 1). The analysis revealed that most of the sequences (~70%) isolated from the urine clustered among each other compared to other samples, regardless of disease status (Figure 1). Additionally, sequences were clustered together based on tissue location, whereas NCCR sequences isolated from the brain and CSF were the most dissimilar (Figure 1). Samples isolated from the blood (~11%, defined in Table 1) clustered to one another and to sequences isolated from the urine, especially from patients diagnosed with HIV or autoimmune diseases such as MS, SLE, or RA, relative to other isolation sites (Figure 1). The clustering among urine samples suggests that the NCCR does not vary greatly in kidneys of healthy individuals. The distant clustering observed in the CSF, brain, and plasma samples observed, reflects genetic rearrangements in the NCCR of diseased patients. Overall, this analysis revealed patterns of sequence similarity among NCCR sequences isolated from various tissues and fluids among PML patients and healthy individuals and the clustering of sequences isolated from the urine and other isolation sites.

### 2.3. The Heterogenicity of NCCR Blocks across Sample Isolation Sites 

Previously published data were used to determine sequence motifs for each block (“a”–“f”) [40]. Initially, the FASTA/Q toolkit, SeqKit, [59] was used to identify the blocks in 181 NCCR sequences from GenBank. Aligned block sequences were manually inspected using Jalview (Version 2.11.0). Then, MEME [60] motifs were generated for the blocks (see Methods) and FIMO [61] was used to map the blocks in all 989 sequences. Next, a Perl script was used to parse the FIMO output and annotate the blocks in the NCCR sequences. A subset of 100 NCCR sequences derived from CSF samples were randomly selected for manual inspection to validate the blocks annotations (see Methods). The total length of the portion of the 989 NCCR sequences covered by blocks had a mean length of 258 bp with a minimum length of 99 bp and maximum length of 523 bp.

To visualize differences in rearrangements of blocks within the 989 NCCR sequences, the occurrence of individual blocks (“a”–“f”) were analyzed to generate NCCR block codes (Table 2). We define NCCR block codes as the arrangement of the individual blocks. Nearly 60% (592) of the 989 NCCR sequences resembled the archetype strain with a block code of ABCDEF. The next most frequently observed NCCR block code, ABCECEF, had a duplication of blocks “c” and “e”. The third most frequent NCCR block code, ABCDCDEF, had a duplication of blocks “c” and “d”. Interestingly, 42% (82/195) of the NCCR sequences from CSF samples from PML patients lacked a block “d”. The archetype NCCR block code was found in sequences from multiple tissues from PML patients. This suggests that the majority of JCPyV genomes may still represent the non-disease strains, and in actuality, a smaller proportion of JCPyV quasispecies, representing a population of duplicated enhancer elements drive replication, and ultimately, lead to disease.

### 2.4. Sequences Isolated from Urine Samples Had a Higher Frequency of TFBS That Repress JCPyV Infection, While TFBS That Facilitate JCPyV Infection Were More Frequent in Sequences from Other Tissues 

**Figure 2:**
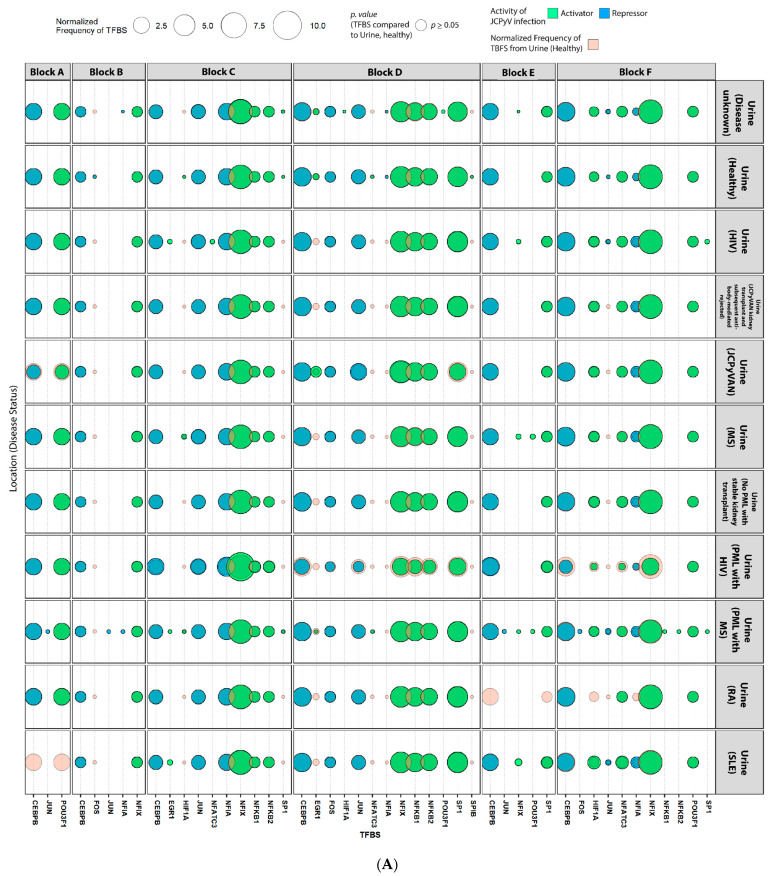

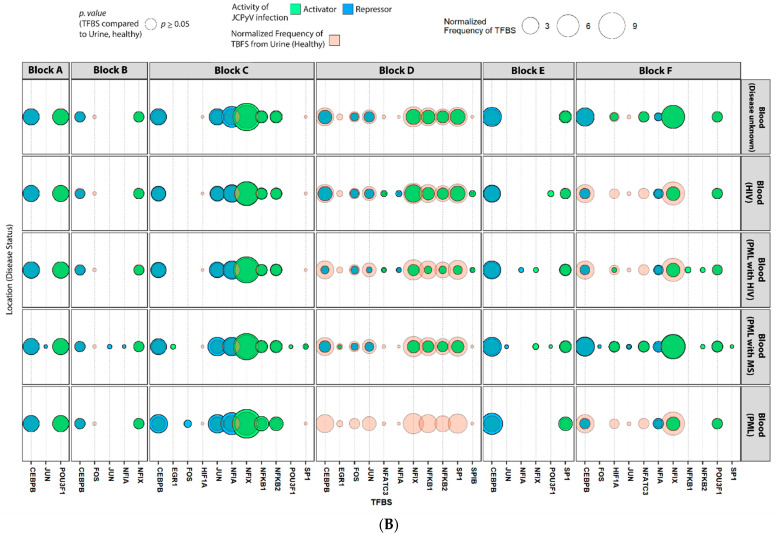

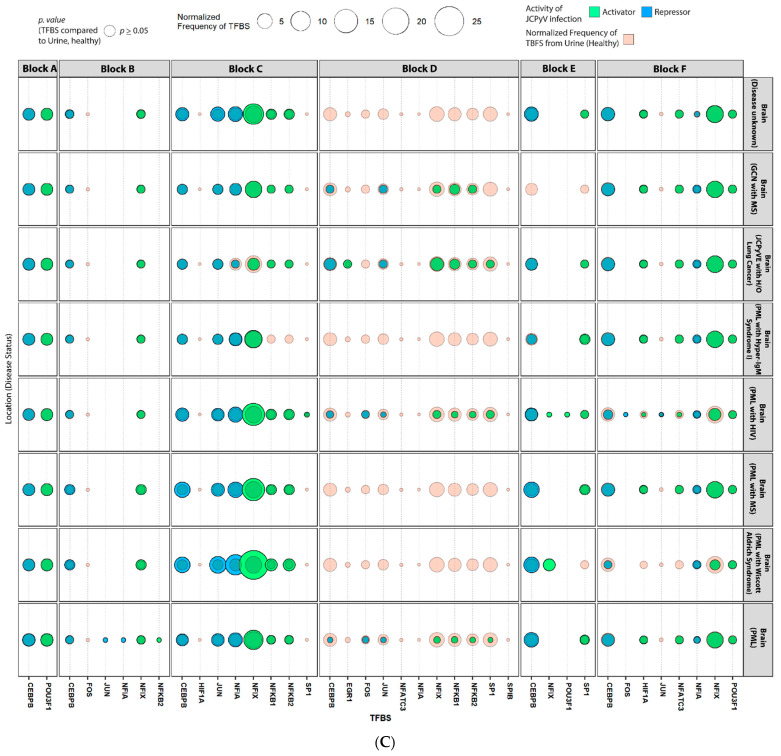

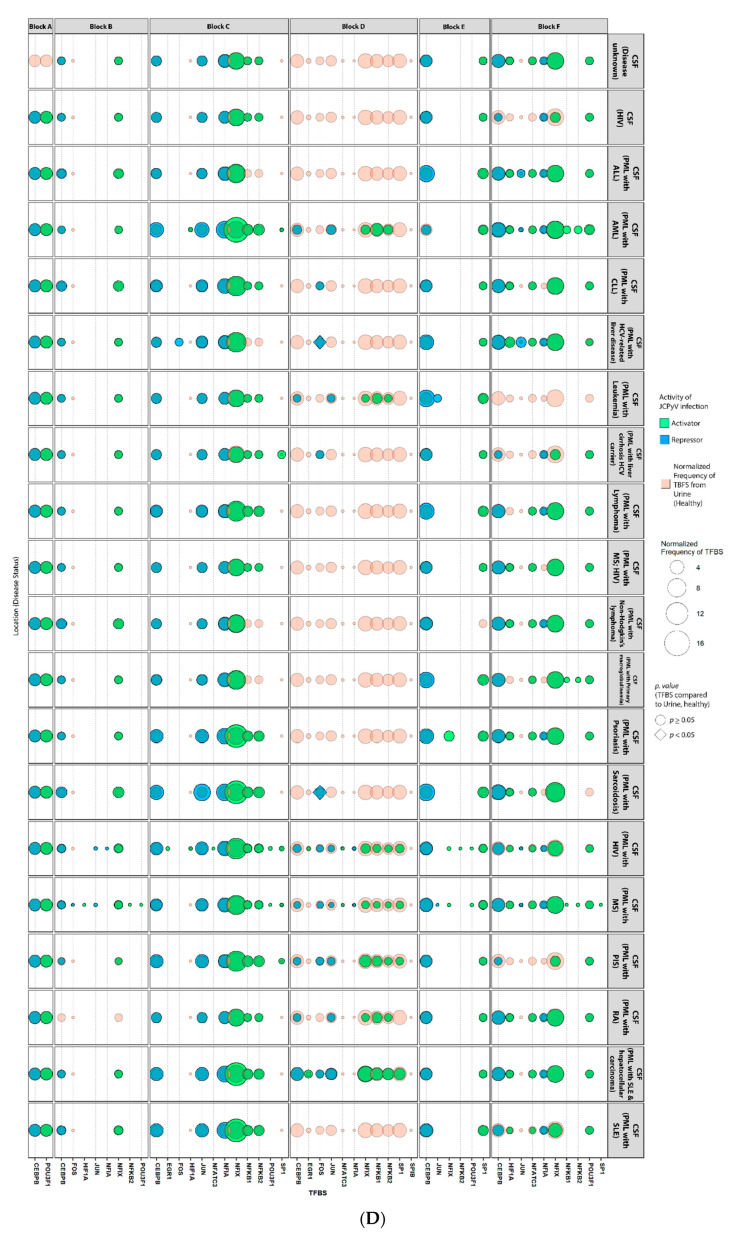
Transcription factors binding sites that are known to activate JCPyV infection are more prevalent in the NCCR of viral isolates from the brain, plasma, and especially, the CSF in patients that have PML or AIDS. The R package, ‘TFBStools’ was used to determine the TFBS for each block, using the 2020 JASPAR database. Known TFBS that have are associated with JCPyV transcription and replication were illustrated as a balloon plot using ‘ggplot2’. TFBS are faceted by the 6 blocks (A–F), disease status, and by tissue source: urine (**A**), blood (**B**), brain (**C**), and CSF (**D**). The size of the shape represents the normalized frequency of the TFBS (i.e., the number of times the TFBS is present) [(# of TFBS/Block)/(# of sequences)] in each of the 6 groups/locations and the color represents the activity that correlates with JCPyV infection. Sequences isolated from the blood represent serum, plasma, and PBMC sequences. Unadjusted *p* values were determined by Fisher’s exact test to compare the normalized frequency of TFBS (size of shapes) from the Urine (Healthy) to all other groups.

To understand how NCCR variation among isolated sequences may influence JCPyV infection, the frequency of known TFBS involved in JCPyV replication from the literature review were determined in each block of every sequence (Figure 2). Sequences from unknown tissue sources and more than one tissue source were excluded in this analysis (*n* = 63). The R package, ‘TFBStools’ (Version 1.26.0) was used along with the 2020 JASPAR database to find the TFBS motifs of each NCCR sequence per block. Sequences isolated from the CSF had the highest number of known TFBS that influence JCPyV infection (Figure 2D). Blocks “c” and “d” had the highest variation in TFBS between sample source, specifically, CCAAT Enhancer Binding Protein β (CEBPβ) and Nuclear Factor I X (NFIX), known repressors and activators of JCPyV infection, respectively (Figure 2). Yet, only the FOS binding site, a repressor of JCPyV infection, occurred more frequently in block “d” of CSF sequences isolated from patients with PML and HCV-related liver disease and patients with PML and Sarcoidosis compared to sequences isolated from the urine of healthy individuals (unadjusted *p* < 0.01, Figure 2D). Interestingly, NFIX sites in block “d”, part of the NCCR that is not an enhancer element [23], were higher in sequences isolated from the urine (Figure 2A). Lastly, the TFBS Spi-B (SPIB) was present in sequences isolated from the blood, specifically in block “d”, adding evidence to the importance of this TF during JCPyV infection of B cells, yet this binding site was not statistically significant (Figure 2B). Overall, the patterns of TFBS in the NCCR sequences support previously-published data on the importance of TFBS in the archetype versus PML-associated strains and how NCCR rearrangements can alter the frequency of TFBS, especially in the brain, plasma, and CSF of PML patients [49,51,57].

### 2.5. Highly Variable Number of TFBS in NCCR Viral Isolates from the Brain, Plasma, and CSF, Specifically in Blocks “c” and “d” 

**Figure 3:**
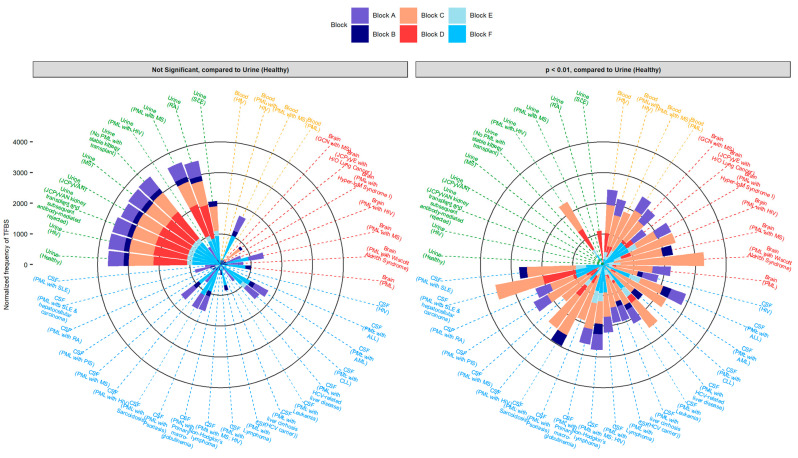
Blocks C, D, and F of the JCPyV NCCR have the largest variation in the frequency of TFBS in sequences isolated from the brain, plasma, and CSF of diseased individuals compared to sequences isolated from the urine of healthy individuals. The overall distribution of the normalized frequency of TFBS [(# of TFBS/Block)/(# of sequences)] were plotted based on disease status and tissue source using the R package, ‘ggplot2’. Adjusted *p* values were determined using either the chi square test adjusting the *p* values using the Bonferroni method or using the fisher exact test, adjusting the *p* values using the Benjamini-Hochberg (BH) method. The selection of these statistical tests was determined by group size and compared the number of normalized frequencies of TFBS (*y*-axis) within each block from the Urine (Healthy) to all other groups.

To determine how rearrangements in the NCCR alter TFBS, especially based on site of viral isolation and disease status, the total number of TFBS were tabulated by disease status and tissue source (Figure 3). A stacked bar graph illustrates the overall number of TFBS in each block of the JCPyV genome. As previously illustrated in Figure 2, the greatest variation in the number of TFBS by location was observed in block “c” and block “d”, specifically in sequences isolated from the CSF (Figure 2D). The frequency of TFBS in block “c” were higher in diseased patients, specifically comparing the number of TFBS from the urine of healthy individuals to the brain and urine of PML patients (adjusted *p* value < 0.01) (Figure 3). Overall, the frequency of TFBS in each block isolated from the urine were less variable and equally distributed compared to nonurine sequences (Figure 3). This is most likely attributed to the duplications of the ‘c’ block and deletions of the ‘d’ block (Table 2). Taken together, these data suggest that the number of TFBS is influenced by the genetic rearrangements in diseased patients, specifically attributed to the duplications and deletions of specific blocks in the NCCR.

### 2.6. The Number of Forkhead and Homeobox TFBS Were Increased in the “c” Block of Patients with PML 

**Figure 4:**
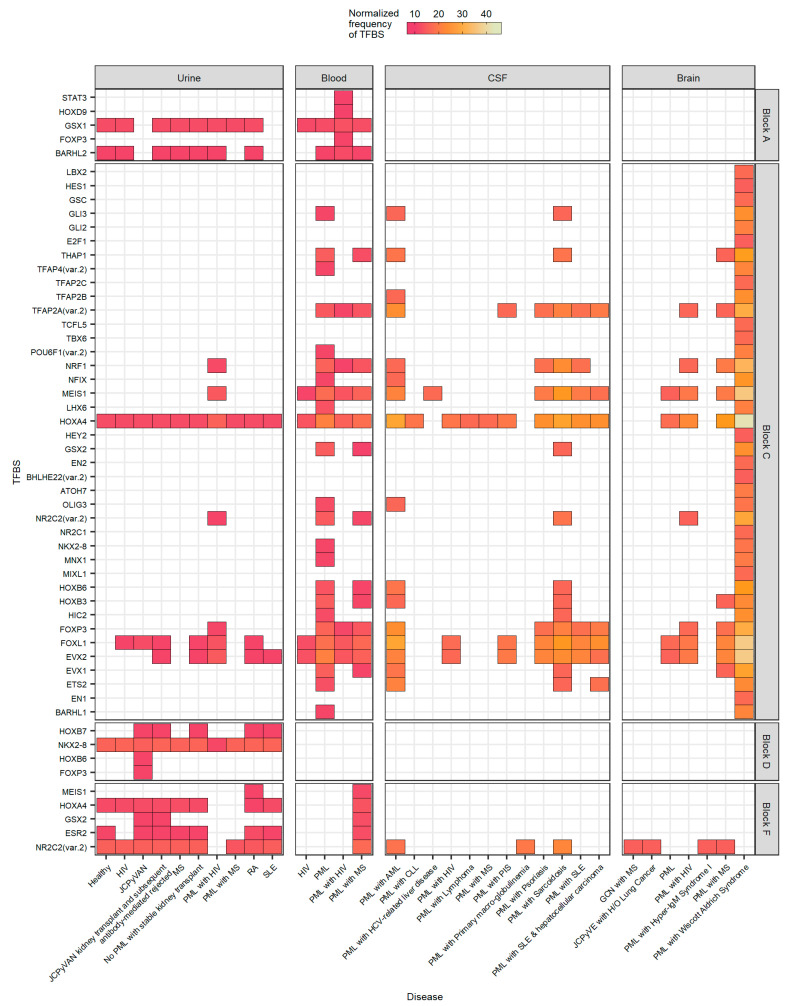
The number and frequency of TFBS in the NCCR, specifically in Block C, are more numerous from nonurine locations of diseased patients. The top 10% of TFBS is illustrated in a heat map, faceted by tissue source and block location. The heat map is colored by the normalized frequency of TFBS in each group.

To understand additional TFBS that are altered in the NCCR sequences of nonurine locations and diseased patients, ~60 TFBS/tissue source representing the top 10% of TFBS were determined (Figure 4). All groups are represented, however not all blocks are represented, as the top 10% of the full sequence was considered. In all, additional TFBS occurred more frequently in PML patients, predominantly occurring in the “c” block (Figure 4). Numerous homeobox protein TFBS were found including HOXA4, MEIS1, and HOXB3, as well as forkhead protein TFBS, such as FOXL1 and FOXP3 (Figure 4). These TFBS, FOXL1 and FOXP3 occurred less frequently in the “d” block in nonurine sequences, specifically within the CSF (*p* = 0.048 and *p* = 0.0062, respectively, data not shown) and more frequently in the “e” block of all nonurine sequences (*p* < 0.05, data not shown). Another TFBS that was found more frequently in PML samples was Nuclear Respiratory Factor 1 (NRF1), a protein-coding gene with DNA-binding transcription activator activity [62,63]. Specifically, the NRF1 binding site was significantly higher in the “c” block of sequences isolated from the CSF compared to sequences isolated from the urine (*p* = 0.0062, data not shown). Most importantly, this analysis also revealed a TFBS for oligodendrocyte transcription factor 3 (OLIG3). This TFBS, which occurred more frequently in the “c” block, may contribute to cellular tropism as it was only observed in sequences isolated from the CSF and brain (Figure 4). However, OLIG3 was only significantly more prevalent in sequences isolated from the blood compared to sequences isolated from the urine in block “c” (*p* = 0.039, data not shown). There were not significant differences when comparing OLIG3 binding sites in the urine to the CSF and brain but it is important to note that another *OLIG* gene member, OLIG2 was also revealed in a similar study [55]. 

The TFBS that occurred less frequently in PML samples were found in block “d”. Specifically, the MEIS1 TFBS had the largest decrease in the number of sites in block “d” in nonurine strains compared to NCCR sequences of urine strains, especially when comparing this TFBS in the urine to the CSF and brain (*p* = 0.0079, data not shown). This may suggest that MEIS1 is important in the development of PML as it was found more frequently in the enhancer element of the JCPyV NCCR. Collectively, this analysis reveals combinations of TFBS that may influence the development of PML, expand the cellular tropism of the virus, and validates TFBS that are known to influence JCPyV infection.

## 3. Discussion

The NCCR is a hypervariable region within the JCPyV genome that can enhance JCPyV infection, and rearrangement of the NCCR is associated with the fatal disease, PML [13]. However, our understanding of how the NCCR is implicated in disease is limited, due in part to low sample sizes [64], and few studies demonstrating the changes in TFBS from sequences isolated from the urine to nonurine locations, and asymptomatic to symptomatic individuals. This study addresses these gaps in knowledge through a metanalysis, compiling 989 NCCR sequences from GenBank to examine how the arrangement of blocks (“a”–“f”) and TFBS found within the blocks were altered across various locations based on individual disease status. Classification of the NCCR sequences using block codes provided a useful representation of the modular duplication, deletion and rearrangement of blocks (“a”–“f”). Our results demonstrate the variability in the NCCR of PML patients and illustrate the differences in the archetype versus PML-type strains of JCPyV. Furthermore, this analysis validates NCCR TFBS previously associated with JCPyV infection and PML while also elucidating TFBS that have not been previously associated with infection and disease.

Sequence analysis revealed that most NCCR sequences isolated from the urine clustered together, however some sequences from diseased patients had nucleotide similarity to sequences isolated from non-urine sources from diseased patients (Figure 1). This was especially true from patients with autoimmune diseases and patients positive for HIV, suggesting that PML-like strains could also be found in the kidney and not restricted to the CNS (Table 2). These same conclusions were also deduced from Pfister et al. [57]. This was contradictory to earlier findings in that significant rearrangement did not occur in the kidney [65,66]. These previous studies could have indicated selection bias and further evidence indicated that mutations, specifically the duplication of the 98 base pair tandem repeat, can occur in the kidneys of PML patients with immunodeficiency disorders [64] (Table 2). These data suggest that even though reactivation of JCPyV is poorly understood, immunosuppressed individuals may not have adequate immune surveillance, prompting enhanced JCPyV replication driven by the probability of promoter rearrangements within the NCCR [13]. However, Figure 1 and Table 2 also illustrate nucleotide similarity and block rearrangement in sequences isolated from the plasma of PML patients comparable to that of sequences isolated from the urine. Lymphocytes can act as reservoirs for JCPyV, and it has been hypothesized that rearrangement of the NCCR can occur here, as B cells have the machinery to facilitate genome rearrangement [24]. Sequences isolated from the plasma illustrated archetype-like structure of the NCCR (Table 2). Taken together, these data demonstrate the likelihood of multiple mechanisms of JCPyV reactivation when comparing sequence identity and block structure of the NCCR in sequences isolated from the urine of healthy individuals versus PML patients.

There have been numerous TFs reported to influence JCPyV infection [24]. However, the impact on the TFBS from rearrangements in the NCCR, in the context of isolation location and disease status, has not been well studied. NFIX is a well characterized TF that activates JCPyV transcription [50,67]. This analysis validates the importance of this TFBS, occurring more frequently in PML patients, specifically, in sequences isolated from the brain and CSF in block “c” of the NCCR (Figure 2B,D). Additionally, this supports the hypothesis that rearrangements in the NCCR increase tissue tropism as NFIX is highly expressed in the CNS, and the addition of these TFBS most likely aids in viral transcription, enhancing replication [50]. Interestingly though, the NFIX TFBS occurred more frequently in sequences isolated from the urine and plasma of PML patients compared to healthy individuals, which demonstrates the importance of NFIX enhancing JCPyV transcription, regardless of location, which may lead to PML.

NCCR mutations are complex and most likely the result of homologous recombination that leads to large deletions and tandem duplications in the NCCR [13] (Table 2). The influence of these rearrangements is also observed in TFBS that are known to influence JCPyV infection (Figure 2). In many sequences isolated from individuals with PML, there are deletions of block “d” (Table 2), which is important in the success of viral transcription as there are TFBS for CEBPβ, a repressor of viral transcription (Figure 2). Deletion of portions or even the full “d” block leads to less TFBS for CEBPβ, which enhances viral transcription [68]. Interestingly however, there were TFBS for NFIX located on the “d” block that occurred more frequently compared to sequences isolated from the brain, plasma, and CSF (Figure 2B–D). This highlights the complexity of not only NCCR rearrangement but also the regulation of TFs in the replication of JCPyV. Romagnoli et al. demonstrated the important inhibitory role of CEBPβ during JCPyV infection of human glioblastoma and transformed human glial cells but also revealed the activation of JCPyV transcription by NF-κB. They suggested that there was a unique interplay between both TFs that control the balance of JCPyV latency and reactivation during immunosuppression [68]. Furthermore, research has also demonstrated that TFs that repress viral transcription, specifically AP-1 TFs, block NF-1 binding sites, because of the proximity of these TFBS [69]. This study isolated human CNS progenitor cells and differentiated them into an astrocytic cell line to determine that AP-1 TFs, like c-Jun, bind to AP-1 binding sites thus blocking TFs that activate transcription as they can no longer bind to NF-1 sites [69]. This analysis revealed that even though there was an increase in NFIX sites in block “d” there was also an increase in TFBS that inhibit JCPyV transcription, like CEPBβ and JUN, which result in lower transcriptional levels that are observed in healthy individuals. These studies examining the importance of TFs in JCPyV infection mainly used in vitro techniques and were across various cell types, and thus it should be noted that cell-type differences can be observed. Overall, examining the known TFBS that influence JCPyV transcription of 989 sequences from healthy and diseased patients reinforced the intricate balance between viral transcription, expanding cellular tropism, and possibly leading to disease caused by enhanced JCPyV replication.

JASPAR is one of the largest databases of known TFBS, and this study examined known TFBS that influence JCPyV infection. In addition to this validation, we further examined other TFBS that have not been previously characterized in JCPyV transcription and cellular tropism, identifying novel TFBS that may correlate with the disease PML (Figure 4). Differences in the number of TFBS were most notable in blocks “c”, “d”, and “f” (Table 2 and Figure 3). This was most likely due to the extent of rearrangements, deletions, and duplications that these blocks are subject to within the NCCR (Table 2). Previous research has also demonstrated significant mutations in the “f” block that resulted in an increase in JCPyV late gene expression [70]. The authors hypothesized this was mostly due to the loss of the TFBS for NFIA and AP1 [70]. Our data support their hypothesis as the TFBS for NFIA was not present in the “f” block, and CEPBβ occurred less frequently, however this did not occur in all disease states (Figure 2B–D). TFBS that occurred more frequently in PML patients, including sequences isolated from the urine, were TFBS associated with HOX gene families (Figure 4). Although HOX genes are important for their role in embryonic development, they have also been implicated in angiogenesis and tumor metastasis [71]. Recently, these genes have also been shown to be involved in hepatitis C virus (HCV) and human cytomegalovirus (HCMV) infection [72,73]. During HCMV infection, numerous HOX genes, including MEIS1, are differentially expressed [72]. A TFBS for MEIS1 was identified in the NCCR of JCPyV across all four isolation sites, yet was most prevalent in the “c” block of nonurine isolates (Figure 4). During HCMV infection, the authors postulated that as TFs, HOX genes regulate downstream gene expression involved in angiogenesis and cellular DNA repair, promoting cell proliferation, inhibiting apoptosis, and possibly being involved in vascular disease pathogenesis [72]. Additionally, these genes are associated with cell cycle-related genes [74]. JCPyV promotes cellular proliferation and inhibits cellular apoptosis through T Ag [26,27] and can influence cyclin expression during the infectious cycle [75]. Perhaps, as viral replication is enhanced by NCCR rearrangements, it may promote further induction of HOX-related genes, which further promote viral transcription; however, molecular studies would need to be performed to validate this hypothesis.

Other TFBS that varied between sequences were binding sites that were associated with FOX genes (Figure 4). The FOX TF family are critical in regulating numerous biological processes, including metabolism, differentiation, proliferation, and apoptosis [76]. Numerous FOX proteins have been demonstrated to be involved in viral infection as well [77]. For example, FOXP3 is upregulated upon human papillomavirus (HPV) infection and may accelerate the cancerous transformation of cervical epithelial cells [77,78,79]. JCPyV can activate cellular pathways, including the mitogen-activated protein kinase, extracellular signal-regulated kinase (MAPK/ERK) pathway [80,81,82,83] and the phosphoinositide 3-kinase/AKT signaling pathway [84,85] that can influence the expression of the FOX TF family, which in turn, may benefit viral transcription, as there are more TFBS in block “c” of the NCCR, isolated from PML patients (Figure 4). Overall, this analysis discovered novel TFBS, specifically related to HOX and FOX TF families that are possibly important in JCPyV replication and disease outcomes. Future studies should validate these findings using molecular and infectivity assays to determine if these TFBS require the binding of TFs to activate JCPyV transcription.

In addition to these findings specific for JCPyV, this study contributes to our understanding of polyomavirus NCCR rearrangements and the role of TFBS in polyomavirus-induced disease. The tripartite genome organization is shared among the 12 identified human polyomaviruses [86] and interestingly, rearrangements in the NCCR correlates with pathogenic disease progression in other polyomaviruses [87]. Notably, BK polyomavirus archetype strain (WW), which infects the kidney and is shed in the urine, is characterized by five sequence blocks: O, P, Q, R, and S [88,89]. The BKPyV NCCR is hypervariable, and detection of the rearranged (rr) strains (i.e., Dunlop), correlates with BKPyV diseases including polyomavirus-associated nephropathy [90,91,92,93]. Further, the BKPyV NCCR contains binding sites for TFs: AP-1, NF-1, NF-kB, Sp1, and Spi-B [89,94,95,96], which are also found in the JCPyV NCCR. Additionally, Sp1 and Spi-B sites are present in all known human polyomaviruses [97]. Overall, this study complements our current knowledge of the similarities among PyV NCCRs and TFBS, which will lead to a better understanding of genetic regulation in polyomavirus reactivation and disease progression.

## 4. Materials and Methods

### 4.1. Determining and Visualizing the Blocks of Each NCCR Viral Isolate

Initially, only 181 sequences were used to obtain the blocks (“a”–“f”) within the NCCR, using already published sequences for each [40]. First, the sequences were separated by isolate site and aligned using Clustal Omega. This step resulted in more accurate block sequences and faster quality control of each NCCR. The blocks were extracted using the command line and the toolkit, SeqKit, an ultrafast FASTA/Q Go programming language [59]. Specifically, the function “locate” was used in the seqkit executable file with varying max mismatch values when matching the known sequence for each block with the 181 sequences, based on the length of nucleotides for the individual blocks (Table 3). The resulting maximum mismatch values provided in Table 3 are the most accurate representation of the NCCR blocks determined by manual inspection. Additionally, only the “+” strand was used. Manual inspection was performed using the program Jalview (Version 2.11.0), an open-source program developed for the editing, analyzing, and visualizing of multiple sequence alignments. This first workflow was later used to validate the Perl script described below to capture more NCCR sequences from GenBank, both, entire NCCR sequences and NCCR sequences extracted from the full JCPyV genome.

Data from the initial smaller subset of NCCR sequences were visualized using the package, ggplot2 (Version 3.3.3) in R and the function geom_pointrange was used to illustrate the position of each block for all the NCCRs faceted by location and disease status. Additionally, the function, geom_histogram was used to determine the density distribution of each block, by length, also faceted by location and disease status (data not shown).

### 4.2. Comprehensive Search for JCPyV NCCR Sequences 

A total of 5507 JCPyV nucleotide sequences were downloaded from the NCBI on 6 December 2021, by searching for all sequences with NCBI Taxonomy ID: 10632. All but one of these sequences were from GenBank. The additional sequence was the complete JCPyV genome sequence for the Mad-1 strain from the Reference Sequence database. Using a custom Perl script, 1049 sequences were identified as NCCR sequences that were less than 700 bp in length and had “control region” or “non-coding regulatory region” listed in the GenBank-formatted sequence records. Of the 1049 sequences, 578 were from one study where multiple sequences were generated from the same tissue source for 17 individuals [34]. The 578 sequences were collapsed to 108 unique sequences for the patient-tissue combinations. Thus, a total of 579 NCCR sequences from the 1049 sequences were included in our analysis. A 554 additional NCCR sequences were extracted from complete JCPyV genome sequence records from set of 5507 sequences using a custom Perl script. All of these genome-derived NCCR sequences began at the ORI site and continued up to the agnoprotein coding sequence. We also required that the ORI site had to be located at position 1 of the assemblies. Of the 554 JCPyV genomes sequences analyzed, 152 of them were from the same study described above [34] and were collapsed to 8 unique sequences based on patient-tissue combinations. Therefore, a total of 410 genome-derived NCCR sequences were included in our analysis. Combining the 579 NCCR sequences together with the 410 genome-derived NCCR sequences gave a total of 989 NCCR sequences that we analyzed.

Sample source information for the 989 NCCR sequences were identified using two steps. First, a Perl script was used to parse the GenBank-formatted sequence records to find source and disease status information in the “note” and “isolation_source” fields or elsewhere in the record and report any PubMed identifiers (IDs). Second, the automatically-parsed information was manually curated to assign a tissue and disease (primary and secondary) status for each sequence. For sequences with PubMed IDs, the publications were reviewed to assign tissue and disease information. For some sequences, publications were not reported in the GenBank-formatted sequence records, but we were able to find the missing publications based on the authors and description in the entries. The curated information for the 989 sequences are in Supplementary Table S1.

### 4.3. Analysis of Blocks among 989 NCCR Sequences

We analyzed the 989 NCCR sequences to find the block A–F motifs using the MEME suite version 5.4.1 [60]. First, MEME motifs were created for blocks A–F using sets of NCCR sequences that manually annotated from CSF samples. A total of 106 block “a”, 156 block “b”, 220 block “c”, 100 block “d”, 175 block “e” and 146 block “f” sequences were used to create one block “a” motif, three block “b” motifs, three block “c” motifs, five block “d” motifs, three block “e” motifs and three block “f” motifs. Locations of these motifs in the NCCR sequences were then mapped using FIMO version 5.4.1 [61]. Custom Perl scripts were then used to filter the output to find matches with q-values < 0.05 and report the matching sequences for the block motifs found in each of the NCCR sequences. A block code was reported as the order of block motifs (e.g., ABCDEF) found in each NCCR sequence.

### 4.4. Validating the Perl Script to Determine the Accuracy and Precision of Capturing the NCCR and the Individual Blocks

To validate the Perl script that was previously mentioned, 100 NCCR sequences isolated from the CSF in the initial analysis were compared with the same sequences captured from the Perl script. Sequences isolated from the CSF were used to validate the script as these sequences had the greatest variation in the NCCR and would serve as a meticulous reference point. The 100 sequences were compared at the block level and at the individual base pairs, the results are summarized in Table 4. Overall, the code was ~90% accurate in representing 85% of the correct block code, determined by the initial analysis (Table 4). The most frequent error occurred at the nucleotide level, yet the sequences were missing an average of 8 base pairs which only constituted of ~3% of the overall NCCR sequence (Table 4).

### 4.5. Cladogram 

The sequences were aligned using Clustal Omega in the command line, using the specific packages and programs, the European Molecular Bioinformatic Open Software Suite (EMBOSS) and ClustalW. Specifically, Clustal 2.1 Multiple Sequence Alignments was used to perform a multiple alignment using the slow and accurate method [58]. Briefly, all sequences were pairwise aligned and a dendrogram was constructed, describing the approximate groupings of the sequences by similarity [58]. Finally, the multiple alignment was carried out, using the dendrogram as a guide. To generate a cladogram, the alignment output was used to calculate the distances between all pairs of sequences. Then the neighbor joining method was used to create the distance matrix [58,98,99]. The .ph file generated from this output was imported into R using the function “read.tree” from the package “ape” (Version 5.4-1). The “phylo4d” function from the package, “phylobase” (Version 0.8.10) was used to merge the cladogram with sequence information, such as where the sequence was isolated from and disease status. Lastly, to visualize the circular cladogram (Figure 1) the function “ggtree” was used from the package, “ggtree” (Version 2.2.4).

### 4.6. Mapping TFBS for Each NCCR Sequence Using the JASPAR Database

To curate the dataset of TFBS for the 1416 blocks, the R packages “TFBStools” (Version 1.26.0) and “Biostrings” (Version 2.56.0) were used to upload the sequences into the RStudio environment with the function “readDNAStringSet”. The matrix for each human TFBS (species ID: 9606) from the 2020 JASPAR database was uploaded into R with the function “getMatrixSet”. TFBS that mapped to the “+” strand of all 989 sequences were predicted using a minimum score of 70% based on the same approach that was used to extract the individual blocks. A minimum score of 70% was determined by analyzing output data with varying scores that resulted in TFBS, such as SPIB and SP1 that are known to influence JCPyV infection, being included in the dataset. To compare the frequency of TFBS with a varying number of sequences from each location, the normalized frequency was determined (Equation (1)).

(1) normalized frequency of TFBS=# of TFBSBlock#of sequences

### 4.7. Statistical Analysis of TFBS

The normalized frequency of TFBS between groups was statistically compared using Fisher’s exact test, adjusting the *p* values using the Benjamini-Hochberg method [100]. For groups with more than 10 sites, statistical significance was determined using the chi-square test, adjuring the *p* values using the Bonferroni method. Lastly, to determine the distribution of the normalized frequency of TFBS in the various tissue sources, the pairwise Wilcoxon rank sum test, along with the Bonferroni adjusted was used to determine the pair of groups that were statistically significant. All analyses were conducted using R version 4.0.5.

R scripts are available upon request. Datasets are provided as supplemental tables, which includes the information for the 989 sequences (Supplemental Table S1) and the frequency of TFBS (Supplemental Table S2).

## 5. Conclusions

**Figure 5:**
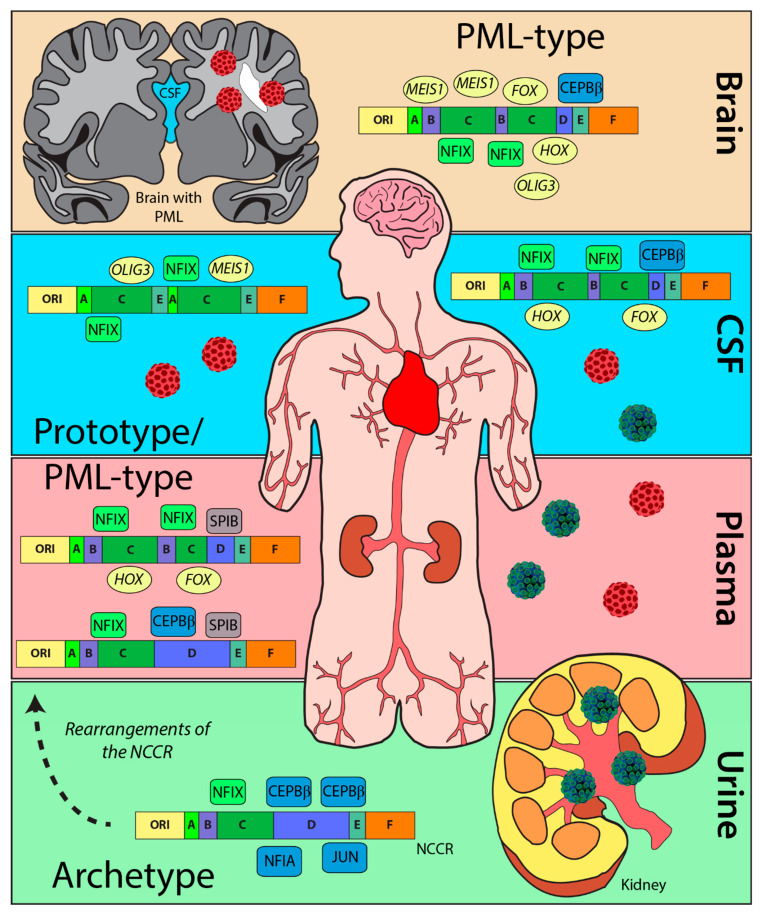
Rearrangements of the NCCR and changes in TFBS. The archetype strain is predominantly detected in the urine of healthy patients (blue/green virus) with numerous TFBS (blue) that inhibit replication. Rearrangements in diseased patients (red virus) causes an increase in TFBS (green) that enhance viral replication of PML-type strains. TFBS, like SPIB (gray) or possibly OLIG3 (yellow), enhance cellular tropism from the rearrangements in the NCCR. Additionally, TFBS, including MEIS1, FOX family transcription factors (*FOX*), and HOX family transcription factors (*HOX*) (yellow) can also be observed from NCCR rearrangements in disease patients compared to TFBS in the NCCR from healthy individuals.

The NCCR of the JCPyV genome is a hypervariable region that is susceptible to complex mutations and rearrangements that are associated with the fatal disease PML. This is one of the first metanalyses that characterize JCPyV NCCR sequences, confirm previously reported TFBS, and identified potential novel TFBS. By curating a dataset of 989 sequences of the JCPyV NCCR, as well as characterizing the block orientation of each sequence, we have illustrated the complexity of this region based on site of viral isolation and disease status of the individual (Figure 5). Additionally, we have validated TFBS that are important in activating or repressing JCPyV transcription by location, disease status, and by each block of the NCCR (Figure 3). Given the effectiveness of this approach, we have discovered possible novel TFBS that correlate with JCPyV infection based on location and disease status (Figure 4). This research contributes to a similar study in which computer simulations were utilized to identify TFBSs in the NCCR [55], leading to new resources for the field and possibly clinicians. Future studies should revisit the outcomes of these TFBS in JCPyV transcription and replication through molecular analyses. To conclude, this research validates and establishes the importance of the NCCR of the JCPyV genome, an understudied area, and in turn, further uncovers the mechanisms of reactivation and PML outcome, which are also poorly understood.

## Figures and Tables

**Table 1:** Summary of the 989 NCCR Sequences.

Tissue Source	Primary Disease State	Total	Secondary Disease	Number of Cases
Brain (*n* = 32)	PML	26 (81%)	HIV	11 (34%)
			MS ^a^	1 (3%)
			WAS ^1^	1 (3%)
			HIGM ^2^	1 (3%)
			N/A	12 (38%)
	JCPyVE ^3^	1 (3%)	H/O Lung Cancer	1 (3%)
	GCN ^b^	1 (3%)	MS	1 (3%)
	N/A	4 (13%)	N/A	4 (13%)
Plasma/serum/PBMC (i.e., blood) (*n* = 111)	PML	91 (82%)	HIV	7 (6%)
			MS	81 (73%)
			N/A	3 (3%)
	Consistent with PML	7 (6%)	HIV	7 (6%)
	N/A	13 (12%)	HIV	5 (5%)
			N/A	8 (7%)
CSF (*n* = 217)	PML	195 (90%)	HIV	46 (21%)
			HIV/MS	2 (1%)
			RA ^4^	1 (1%)
			SLE ^c^	9 (4%)
			MS	80 (37%)
			AML ^d^	15 (7%)
			ALL ^e^	7 (3%)
			CLL ^f^	4 (2%)
			NHL	4 (2%)
			WM ^5^	8 (4%)
			Other ^#^	14 (6%)
			N/A	5 (2%)
	Consistent with PML	6 (3%)	HIV	6 (3%)
	Suspected of PML	14 (6%)	HIV	2 (1%)
			MS	1 (1%)
			N/A	11 (5%)
	N/A	2 (1%)	HIV	1 (1%)
			N/A	1 (1%)
Kidney (*n* = 2)	JCPyVAN ^6^	2 (100%)	N/A	2 (100%)
Kidney; Urine (*n* = 3)	JCPyVAN ^6^	3 (100%)	N/A	3 (100%)
Brain; Kidney (*n* = 6)	N/A	6 (100%)	N/A	6 (100%)
CSF; Plasma (*n* = 2)	Consistent with PML	2 (100%)	HIV	2 (100%)
Urine (*n* = 565)	PML	78 (14%)	HIV	4 (1%)
			MS	74 (13%)
	JCPyVAN ^6^	1 (0%)	N/A	1 (0%)
		4 (1%)	kidney transplant and subsequent antibody-mediated rejection	4 (1%)
	Healthy	179 (15%)	N/A	179 (32%)
	No PML	25 (%)	Stable Kidney Transplant	25 (4%)
	N/A	279 (65%)	HIV	21 (4%)
			SLE	8 (1%)
			MS	12 (2%)
			RA	1 (0%)
			N/A	236 (42%)
No tissue reported (*n* = 50)%	PML	47 (94%)	HIV	3 (6%)
			MS	44 (88%)
	N/A	3 (6%)	N/A	3 (6%)

^1^ Wiscott Aldrich syndrome; ^2^ Hyper IgM syndrome; ^3^ JC Virus encephalopathy; ^4^ Rheumatoid arthritis; ^5^ Waldenstrom macroglobulinemia; ^6^ JC Virus-associated nephropathy; ^a^ Multiple sclerosis; ^b^ Granule cell neuronopathy; ^c^ Systemic lupus erythematosus; ^d^ Acute myeloid leukemia; ^e^ Acute lymphoblastic leukemia; ^f^ Chronic lymphocytic leukemia; # Other include: Primary Immunodeficiency Syndrome (3); Sarcoidosis (3); Psoriasis (1); Leukemia (1); Lymphoma (1); HCV-related liver disease (5);% HG764413 has plasma, urine, CSF and kidney listed as the tissue.

**Table 2:** Summary of the NCCR Block Codes by Tissue and PML Disease Status.

NCCR Block Code	Total #	Number of PML Patient Samples (Total Samples)					
		CSF	Urine	Blood	Brain	Other	Not Specified
AB----CD-----------------EF	592	35 (36)	75 (490)	35 (44)	1 (2)	0 (6)	13 (14)
AB----C—-E-------------C-EF	74	20 (23)	1 (1)	36 (38)	2 (4)	0 (1)	7 (7)
AB----CD---------------CDEF	64	13 (13)	2 (50)			0 (1)	
AB----C—-E--B----------C-EF	28	19 (22)	0 (1)		2 (3)		2 (2)
AB----C------------------EF	22	9 (14)		5 (6)	2 (2)		
AB----C—-E------------FC-EF	21	9 (9)		10 (10)	1 (2)		
AB----CD-E—-B----------CDEF	17	9 (10)		0 (1)	2 (2)		4 (4)
A-----CD-----------------EF	13	2 (2)	0 (10)	0 (1)			
AB----CD-E-------------CDEF	11	6 (7)		0 (1)	2 (2)	0 (1)	
AB----CD-----CD------EFCDEF	10	10 (10)					
AB----C-------------------F	10	7 (7)					3 (3)
AB----CD-EF-B----------CDEF	10	5 (5)					5 (5)
AB----C-B--------------C-EF	10	2 (2)		0 (1)		0 (2)	5 (5)
A-----C—-E-A-----------C-EF	9	2 (4)		2 (2)	2 (2)	0 (1)	
-B----CD-----------------EF	8		0 (8)				
AB----CD-------------EFCDEF	7	5 (5)			1 (1)		1 (1)
AB----C-B--------------CDEF	5	4 (5)					
AB----CD-----------------E-	4	3 (3)	0 (1)				
A-----CD---------------CDEF	4	2 (2)	0 (2)				
AB----CD------------------F	4	1 (2)	0 (1)		0 (1)		
A-----C------------------EF	4	1 (1)		0 (2)		0 (1)	
AB----C-B----C-E-------C-EF	4						4 (4)
AB----C-B----CD--------CDEF	3	3 (3)					
AB----CDB--------------CDEF	3	3 (3)					
AB----CD—------------EF-DEF	3	1 (1)		1 (1)	1 (1)		
A-----C—-E-------------C-EF	3	2 (2)		0 (1)			
AB----CD---------------CDE-	3	1 (1)		1 (1)	1 (1)		
AB----C--E--BC-E-B-----C-EF	2	2 (2)					
AB----C--EF-BC-E-B-----C-EF	2	2 (2)					
AB----C-B----C---B-----C-EF	2	1 (1)					0 (1)
AB----CD-------------EFC--F	2						2 (2)
AB----C------------------E-	2			1 (1)			1 (1)
AB----CD-----CDEFB-----CDEF	1	1 (1)					
AB----CD-----CDEF--CDEFCDEF	1	1 (1)					
AB----CD-E--B----------CDE-	1	1 (1)					
AB----CD-EF-BCDEFB-----CDEF	1	1 (1)					
AB----CD-EF--CDEF--CDEFCD--	1	1 (1)					
AB----C--E-A-----B-----C-EF	1	1 (1)					
AB----C--E---C---B-----C-EF	1	1 (1)					
AB----C--E---C-E-B-----C-EF	1	1 (1)					
AB----C--E---C-E---C-E-C-EF	1	1 (1)					
AB----C--E---C-------EFC-EF	1	1 (1)					
AB-E--C--E---C-------E-C-EF	1	1 (1)					
AB---FCD-----------------EF	1	1 (1)					
A----FCD-----------------EF	1	1 (1)					
-B----CD-------------E-CDEF	1	1 (1)					
AB----C--E-------------C--F	1	0 (1)					
AB----C--EF-B----------C-EF	1	0 (1)					
A----FCD---------------CDEF	1	0 (1)					
-BC----------------------EF	1	0 (1)					
AB----CD----B----------C--F	1				1 (1)		
AB----CD---------------CD-F	1				1 (1)		
AB----CD-E--BC-E-B-----CDEF	1				1 (1)		
AB----CD-EF--------CDEFCDEF	1				1 (1)		
AB----C--E---C---B-C-E-C-EF	1				1 (1)		
AB----C--E---------C-E-C-EF	1				1 (1)		
A-----CD---------------CD-F	1				1 (1)		
ABC-B-CD----BCD--B-----C---	1	1 (1)					
A-----C------------------E-	1				1 (1)		
AB----CD-----------CD-F--E-	1				1 (1)		
AB----CD-------------EF--EF	1		0 (1)				
AB-----D-----------------EF	1		0 (1)				
AB----C--E-A-----------C-EF	1			0 (1)			
A-----CD----B----------CDEF	1	1 (1)					
A-----C--E-A-C-E--A----C-EF	1					0 (1)	
**Total**	**989**	**195 (217)**	**78 (566)**	**91 (111)**	**26 (32)**	**0 (14)**	**47 (49)**

# = number of sequences analyzed.

**Table 3:** Block sequences and criteria used to locate them in each sequence.

Block Letter	Nucleotide Sequence	Maximum Mismatch Value
Block “a”	CCTGTATATATAAAAAAAAGGGAAGG	9
Block “b”	AGGGAGGAGCTGGCTAAAACTG	8
Block “c”	GATGGCTGCCAGCCAAGCATGAGCTCATACCTAGGGAGCCAACCAGCTGACAGCC	27
Block “d”	AGAGGGAGCCCTGGCTGCATGCCACTGGCAGTTATAGTGAAACCCCTCCCATAGTCCTTAATCACA	31
Block “e”	AGTAAACAAAGCACAAGG	1
Block “f”	GGAAGTGGAAAGCAGCCAAGGGAACATGTTTTGCGAGCCAGAGCTGTTTTGGCTTGTCACCAGCTGGCCAGT	31

**Table 4:** Summary comparing 100 NCCR sequences from the automated script to the initial, manual analysis.

Description of Error	Frequency of Error (%)	Mean (SD) (Block Code or Base Pairs)	% of Error out of the Average Length of the NCCR with Error (SD)
Block code less than 85% accurate	13%	77.7% (±4.2%)	N/A
Block code greater than 100% (larger blocks from the initial analysis were interpreted as smaller and different block codes)	6%	123.5% (±26%)	N/A
Autogenerated block annotation had nucleotides counted in two sequential blocks, occurring when the start of a block occurs before the end of the previous	63%	2.93 (±1.88)	0.998% (±0.0807%)
Predicted block lacks at least one base pair that was counted in the manual annotation	93%	8.84 (±9.12)	3.07% (±3.29%)

## Data Availability

The data presented in this study are publicly available and scripts are available on request from the corresponding authors.

## Supplementary Materials

The following are available online at https://www.mdpi.com/article/10.3390/ijms23105699/s1.

## References

[1] Kean J.M., Rao S., Wang M., Garcea R.L. (2009). Seroepidemiology of Human Polyomaviruses. PLoS Pathog..

[2] Egli A., Infanti L., Dumoulin A., Buser A., Samaridis J., Stebler C., Gosert R., Hirsch H.H. (2009). Prevalence of Polyomavirus BK and JC Infection and Replication in 400 Healthy Blood Donors. J. Infect. Dis..

[3] Hirsch H.H., Kardas P., Kranz D., Leboeuf C. (2013). The Human JC Polyomavirus (JCPyV): Virological Background and Clinical Implications. APMIS.

[4] Padgett B.L., Walker D.L., ZuRhein G.M., Eckroade R.J., Dessel B.H. (1971). Cultivation of Papova-like Virus from Human Brain with Progressive Multifocal Leucoencephalopathy. Lancet.

[5] Monaco M.C., Atwood W.J., Gravell M., Tornatore C.S., Major E.O. (1996). JC Virus Infection of Hematopoietic Progenitor Cells, Primary B Lymphocytes, and Tonsillar Stromal Cells: Implications for Viral Latency. J. Virol..

[6] Dubois V., Dutronc H., Lafon M.E., Poinsot V., Pellegrin J.L., Ragnaud J.M., Ferrer A.M., Fleury H.J. (1997). Latency and Reactivation of JC Virus in Peripheral Blood of Human Immunodeficiency Virus Type 1-Infected Patients. J. Clin. Microbiol..

[7] Chapagain M.L., Nerurkar V.R. (2010). Human Polyomavirus JC (JCV) Infection of Human B Lymphocytes: A Possible Mechanism for JCV Transmigration across the Blood-Brain Barrier. J. Infect. Dis..

[8] White M.K., Khalili K. (2011). Pathogenesis of Progressive Multifocal Leukoencephalopathy—Revisited. J. Infect. Dis..

[9] Pavlovic D., Patera A.C., Nyberg F., Gerber M., Liu M., Progressive Multifocal Leukeoncephalopathy Consortium (2015). Progressive Multifocal Leukoencephalopathy: Current Treatment Options and Future Perspectives. Adv. Neurol. Disord..

[10] Tan I.L., Koralnik I.J., Rumbaugh J.A., Burger P.C., King-Rennie A., McArthur J.C. (2011). Progressive Multifocal Leukoencephalopathy in a Patient without Immunodeficiency. Neurology.

[11] Albrecht H., Hoffmann C., Degen O., Stoehr A., Plettenberg A., Mertenskötter T., Eggers C., Stellbrink H.-J. (1998). Highly Active Antiretroviral Therapy Significantly Improves the Prognosis of Patients with HIV-Associated Progressive Multifocal Leukoencephalopathy. Aids.

[12] Engsig F.N., Hansen A.-B.E., Omland L.H., Kronborg G., Gerstoft J., Laursen A.L., Pedersen C., Mogensen C.B., Nielsen L., Obel N. (2009). Incidence, Clinical Presentation, and Outcome of Progressive Multifocal Leukoencephalopathy in HIV-Infected Patients during the Highly Active Antiretroviral Therapy Era: A Nationwide Cohort Study. J. Infect. Dis..

[13] Cortese I., Reich D.S., Nath A. (2021). Progressive Multifocal Leukoencephalopathy and the Spectrum of JC Virus-Related Disease. Nat. Rev. Neurol..

[14] Carson K.R., Evens A.M., Richey E.A., Habermann T.M., Focosi D., Seymour J.F., Laubach J., Bawn S.D., Gordon L.I., Winter J.N. (2009). Progressive Multifocal Leukoencephalopathy after Rituximab Therapy in HIV-Negative Patients: A Report of 57 Cases from the Research on Adverse Drug Events and Reports Project. Blood.

[15] Bloomgren G., Richman S., Hotermans C., Subramanyam M., Goelz S., Natarajan A., Lee S., Plavina T., Scanlon J.V., Sandrock A. (2012). Risk of Natalizumab-Associated Progressive Multifocal Leukoencephalopathy. N. Engl. J. Med..

[16] Vermersch P., Kappos L., Gold R., Foley J.F., Olsson T., Cadavid D., Bozic C., Richman S. (2011). Clinical Outcomes of Natalizumab-Associated Progressive Multifocal Leukoencephalopathy (Podcast). Neurology.

[17] Prosperini L., de Rossi N., Scarpazza C., Moiola L., Cosottini M., Gerevini S., Capra R., on behalf of the Italian PML study group (2016). Natalizumab-Related Progressive Multifocal Leukoencephalopathy in Multiple Sclerosis: Findings from an Italian Independent Registry. PLoS ONE.

[18] Balduzzi A., Lucchini G., Hirsch H.H., Basso S., Cioni M., Rovelli A., Zincone A., Grimaldi M., Corti P., Bonanomi S. (2011). Polyomavirus JC-Targeted T-Cell Therapy for Progressive Multiple Leukoencephalopathy in a Hematopoietic Cell Transplantation Recipient. Bone Marrow Transpl..

[19] Muftuoglu M., Olson A., Marin D., Ahmed S., Mulanovich V., Tummala S., Chi T.L., Ferrajoli A., Kaur I., Li L. (2018). Allogeneic BK Virus–Specific T Cells for Progressive Multifocal Leukoencephalopathy. N. Engl. J. Med..

[20] Cortese I., Muranski P., Enose-Akahata Y., Ha S.-K., Smith B., Monaco M., Ryschkewitsch C., Major E.O., Ohayon J., Schindler M.K. (2019). Pembrolizumab Treatment for Progressive Multifocal Leukoencephalopathy. N. Engl. J. Med..

[21] Assetta B., Atwood W.J. (2017). The Biology of JC Polyomavirus. Biol. Chem..

[22] Liddington R.C., Yan Y., Moulai J., Sahli R., Benjamin T.L., Harrison S.C. (1991). Structure of Simian Virus 40 at 3.8-A Resolution. Nature.

[23] Frisque R.J., Bream G.L., Cannella M.T. (1984). Human Polyomavirus JC Virus Genome. J. Virol..

[24] Ferenczy M.W., Marshall L.J., Nelson C.D., Atwood W.J., Nath A., Khalili K., Major E.O. (2012). Molecular Biology, Epidemiology, and Pathogenesis of Progressive Multifocal Leukoencephalopathy, the JC Virus-Induced Demyelinating Disease of the Human Brain. Clin. Microbiol. Rev..

[25] Vogelstein B., Lane D., Levine A.J. (2000). Surfing the P53 Network. Nature.

[26] Dyson N., Bernards R., Friend S.H., Gooding L.R., Hassell J.A., Major E.O., Pipas J.M., Vandyke T., Harlow E. (1990). Large T Antigens of Many Polyomaviruses Are Able to Form Complexes with the Retinoblastoma Protein. J. Virol..

[27] Valle L.D., Gordon J., Assimakopoulou M., Enam S., Geddes J.F., Varakis J.N., Katsetos C.D., Croul S., Khalili K. (2001). Detection of JC Virus DNA Sequences and Expression of the Viral Regulatory Protein T-Antigen in Tumors of the Central Nervous System. Cancer Res..

[28] Dickmanns A., Zeitvogel A., Simmersbach F., Weber R., Arthur A.K., Dehde S., Wildeman A.G., Fanning E. (1994). The Kinetics of Simian Virus 40-Induced Progression of Quiescent Cells into S Phase Depend on Four Independent Functions of Large T Antigen. J. Virol..

[29] Khalili K., Sariyer I.K., Safak M. (2008). Small Tumor Antigen of Polyomaviruses: Role in Viral Life Cycle and Cell Transformation. J. Cell. Physiol..

[30] Bollag B., Kilpatrick L.H., Tyagarajan S.K., Tevethia M.J., Frisque R.J. (2006). JC Virus T’135, T’136 and T’165 Proteins Interact with Cellular P107 and P130 in Vivo and Influence Viral Transformation Potential. J. Neurovirol..

[31] Chen X.S., Stehle T., Harrison S.C. (1998). Interaction of Polyomavirus Internal Protein VP2 with the Major Capsid Protein VP1 and Implications for Participation of VP2 in Viral Entry. EMBO J..

[32] Safak M., Barrucco R., Darbinyan A., Okada Y., Nagashima K., Khalili K. (2001). Interaction of JC Virus Agno Protein with T Antigen Modulates Transcription and Replication of the Viral Genome in Glial Cells. J. Virol..

[33] Suzuki T., Orba Y., Okada Y., Sunden Y., Kimura T., Tanaka S., Nagashima K., Hall W.W., Sawa H. (2010). The Human Polyoma JC Virus Agnoprotein Acts as a Viroporin. PLoS Pathog..

[34] Reid C.E., Li H., Sur G., Carmillo P., Bushnell S., Tizard R., McAuliffe M., Tonkin C., Simon K., Goelz S. (2011). Sequencing and Analysis of JC Virus DNA from Natalizumab-Treated PML Patients. J. Infect. Dis..

[35] Agostini H.T., Ryschkewitsch C.F., Singer E.J., Stoner G.L. (1997). JC Virus Regulatory Region Rearrangements and Genotypes in Progressive Multifocal Leukoencephalopathy: Two Independent Aspects of Virus Variation. J. Gen. Virol..

[36] White M.K., Safak M., Khalili K. (2009). Regulation of Gene Expression in Primate Polyomaviruses. J. Virol..

[37] Sabath B.F., Major E.O. (2002). Traffic of JC Virus from Sites of Initial Infection to the Brain: The Path to Progressive Multifocal Leukoencephalopathy. J. Infect. Dis..

[38] Marshall L.J., Major E.O. (2010). Molecular Regulation of JC Virus Tropism: Insights into Potential Therapeutic Targets for Progressive Multifocal Leukoencephalopathy. J. Neuroimmune Pharmacol. Off. J. Soc. NeuroImmune Pharmacol..

[39] Yogo Y., Kitamura T., Sugimoto C., Ueki T., Aso Y., Hara K., Taguchi F. (1990). Isolation of a Possible Archetypal JC Virus DNA Sequence from Nonimmunocompromised Individuals. J. Virol..

[40] Ault G.S., Stoner G.L. (1993). Human Polyomavirus JC Promoter/Enhancer Rearrangement Patterns from Progressive Multifocal Leukoencephalopathy Brain Are Unique Derivatives of a Single Archetypal Structure. J. Gen. Virol..

[41] Jensen P.N., Major E.O. (2001). A Classification Scheme for Human Polyomavirus JCV Variants Based on the Nucleotide Sequence of the Noncoding Regulatory Region. J. Neurovirology.

[42] Loy T.V., Thys K., Ryschkewitsch C., Lagatie O., Monaco M.C., Major E.O., Tritsmans L., Stuyver L.J. (2015). JC Virus Quasispecies Analysis Reveals a Complex Viral Population Underlying Progressive Multifocal Leukoencephalopathy and Supports Viral Dissemination via the Hematogenous Route. J. Virol..

[43] Ciardi M.R., Zingaropoli M.A., Iannetta M., Prezioso C., Perri V., Pasculli P., Lichtner M., d’Ettorre G., Altieri M., Conte A. (2020). JCPyV NCCR Analysis in PML Patients with Different Risk Factors: Exploring Common Rearrangements as Essential Changes for Neuropathogenesis. Virol. J..

[44] Martin J.D., King D.M., Slauch J.M., Frisque R.J. (1985). Differences in Regulatory Sequences of Naturally Occurring JC Virus Variants. J. Virol..

[45] Martin J.D., Foster G.C. (1984). Multiple JC Virus Genomes from One Patient. J. Gen. Virol..

[46] Frisque R.J. (1983). Nucleotide Sequence of the Region Encompassing the JC Virus Origin of DNA Replication. J. Virol..

[47] Daniel A.M., Swenson J.J., Mayreddy R.P.R., Khalili K., Frisque R.J. (1996). Sequences within the Early and Late Promoters of Archetype JC Virus Restrict Viral DNA Replication and Infectivity. Virology.

[48] Gronostajski R.M. (2000). Roles of the NFI/CTF Gene Family in Transcription and Development. Gene.

[49] Amemiya K., Traub R., Durham L., Major E.O. (1992). Adjacent Nuclear Factor-1 and Activator Protein Binding Sites in the Enhancer of the Neurotropic JC Virus. A Common Characteristic of Many Brain-Specific Genes. J. Biol. Chem..

[50] Sumner C., Shinohara T., Durham L., Traub R., Major E.O., Amemiya K. (1996). Expression of Multiple Classes of the Nuclear Factor-1 Family in the Developing Human Brain: Differential Expression of Two Classes of NF-1 Genes. J. Neurovirol..

[51] Marshall L.J., Dunham L., Major E.O. (2010). Transcription Factor Spi-B Binds Unique Sequences Present in the Tandem Repeat Promoter/Enhancer of JC Virus and Supports Viral Activity. J. Gen. Virol..

[52] Manley K., O’hara B.A., Gee G.V., Simkevich C.P., Sedivy J.M., Atwood W.J. (2006). NFAT4 Is Required for JC Virus Infection of Glial Cells. J. Virol..

[53] Marshall L.J., Ferenczy M.W., Daley E.L., Jensen P.N., Ryschkewitsch C.F., Major E.O. (2014). Lymphocyte Gene Expression and JC Virus Noncoding Control Region Sequences Are Linked with the Risk of Progressive Multifocal Leukoencephalopathy. J. Virol..

[54] Frohman E.M., Monaco M.C., Remington G., Ryschkewitsch C., Jensen P.N., Johnson K., Perkins M., Liebner J., Greenberg B., Monson N. (2014). JC Virus in CD34^+^ and CD19^+^ Cells in Patients with Multiple Sclerosis Treated with Natalizumab. JAMA Neurol..

[55] Nakamichi K., Shimokawa T. (2021). Database and Statistical Analyses of Transcription Factor Binding Sites in the Non-Coding Control Region of JC Virus. Viruses.

[56] Fornes O., Castro-Mondragon J.A., Khan A., van der Lee R., Zhang X., Richmond P.A., Modi B.P., Correard S., Gheorghe M., Baranašić D. (2019). JASPAR 2020: Update of the Open-Access Database of Transcription Factor Binding Profiles. Nucleic Acids Res..

[57] Pfister L.-A., Letvin N.L., Koralnik I.J. (2001). JC Virus Regulatory Region Tandem Repeats in Plasma and Central Nervous System Isolates Correlate with Poor Clinical Outcome in Patients with Progressive Multifocal Leukoencephalopathy. J. Virol..

[58] Larkin M.A., Blackshields G., Brown N.P., Chenna R., McGettigan P.A., McWilliam H., Valentin F., Wallace I.M., Wilm A., Lopez R. (2007). Clustal W and Clustal X Version 2.0. Bioinformatics.

[59] Shen W., Le S., Li Y., Hu F. (2016). SeqKit: A Cross-Platform and Ultrafast Toolkit for FASTA/Q File Manipulation. PLoS ONE.

[60] Bailey T.L., Johnson J., Grant C.E., Noble W.S. (2015). The MEME Suite. Nucleic Acids Res..

[61] Grant C.E., Bailey T.L., Noble W.S. (2011). FIMO: Scanning for Occurrences of a given Motif. Bioinformatics.

[62] Scarpulla R.C. (2008). Nuclear Control of Respiratory Chain Expression by Nuclear Respiratory Factors and PGC-1-Related Coactivator. Ann. N. Y. Acad. Sci..

[63] Dhar S.S., Wong-Riley M.T.T. (2009). Coupling of Energy Metabolism and Synaptic Transmission at the Transcriptional Level: Role of Nuclear Respiratory Factor 1 in Regulating Both Cytochrome c Oxidase and NMDA Glutamate Receptor Subunit Genes. J. Neurosci..

[64] Boldorini R., Omodeo-Zorini E., Nebuloni M., Benigni E., Vago L., Ferri A., Monga G. (2003). Lytic JC Virus Infection in the Kidneys of AIDS Subjects. Mod. Pathol..

[65] Inaga T.T., Yogo Y., Kitamura T., Aso Y. (1992). Persistence of Archetypal JC Virus DNA in Normal Renal Tissue Derived from Tumor-Bearing Patients. Virology.

[66] Agostini H.T., Ryschkewitsch C.F., Stoner G.L. (1998). Rearrangements of Archetypal Regulatory Regions in JC Virus Genomes from Urine. Res. Virol..

[67] Shinohara T., Nagashima K., Major E.O. (1997). Propagation of the Human Polyomavirus, JCV, in Human Neuroblastoma Cell Lines. Virology.

[68] Romagnoli L., Wollebo H.S., Deshmane S.L., Mukerjee R., Valle L.D., Safak M., Khalili K., White M.K. (2009). Modulation of JC Virus Transcription by C/EBPbeta. Virus Res..

[69] Ravichandran V., Sabath B.F., Jensen P.N., Houff S.A., Major E.O. (2006). Interactions between C-Jun, Nuclear Factor 1, and JC Virus Promoter Sequences: Implications for Viral Tropism. J. Virol..

[70] L’Honneur A.-S.S., Leh H., Laurent-Tchenio F., Hazan U., Rozenberg F., Bury-Moné S. (2018). Exploring the Role of NCCR Variation on JC Polyomavirus Expression from Dual Reporter Minicircles. PLoS ONE.

[71] Li B., Huang Q., Wei G.-H. (2019). The Role of HOX Transcription Factors in Cancer Predisposition and Progression. Cancers.

[72] Li L., Liu M., Kang L., Li Y., Dai Z., Wang B., Liu S., Chen L., Tan Y., Wu G. (2016). HHEX: A Crosstalker between HCMV Infection and Proliferation of VSMCs. Front. Cell. Infect. Microbiol..

[73] Kasai H., Mochizuki K., Tanaka T., Yamashita A., Matsuura Y., Moriishi K. (2020). Induction of HOX Genes by Hepatitis C Virus Infection via Impairment of Histone H2A Monoubiquitination. J. Virol..

[74] Liu W.-J., Zhang T., Guo Q.-L., Liu C.-Y., Bai Y.-Q. (2016). Effect of ATRA on the Expression of HOXA5 Gene in K562 Cells and Its Relationship with Cell Cycle and Apoptosis. Mol. Med. Rep..

[75] Wilczek M.P., DuShane J.K., Armstrong F.J., Maginnis M.S. (2019). JC Polyomavirus Infection Reveals Delayed Progression of the Infectious Cycle in Normal Human Astrocytes. J. Virol..

[76] Golson M.L., Kaestner K.H. (2016). Fox Transcription Factors: From Development to Disease. Development.

[77] Ramezani A., Nikravesh H., Faghihloo E. (2019). The Roles of FOX Proteins in Virus-associated Cancers. J. Cell. Physiol..

[78] Zeng C., Yao Y., Jie W., Zhang M., Hu X., Zhao Y., Wang S., Yin J., Song Y. (2013). Up-Regulation of Foxp3 Participates in Progression of Cervical Cancer. Cancer Immunol. Immunother..

[79] Mitildzans A., Isajevs S., Rezeberga D. (2019). P33 Up-Regulation of FOXP3 T Regulatory Lymphocytes in Patients with High-Grade Squamous Intraepithelial Lesions Correlated with HPV Infection. Int. J. Gynecol. Cancer.

[80] DuShane J.K., Wilczek M.P., Mayberry C.L., Maginnis M.S. (2018). ERK Is a Critical Regulator of JC Polyomavirus Infection. J. Virol..

[81] Shaul Y.D., Seger R. (2007). The MEK/ERK Cascade: From Signaling Specificity to Diverse Functions. Biochim. Biophys. Acta BBA-Mol. Cell. Res..

[82] Querbes W., Benmerah A., Tosoni D., Fiore P.P.D., Atwood W.J. (2003). A JC Virus-Induced Signal Is Required for Infection of Glial Cells by a Clathrin- and Eps15-Dependent Pathway. J. Virol..

[83] Wilczek M.P., Armstrong F.J., Geohegan R.P., Mayberry C.L., DuShane J.K., King B.L., Maginnis M.S. (2021). The MAPK/ERK Pathway and the Role of DUSP1 in JCPyV Infection of Primary Astrocytes. Viruses.

[84] Peterson J.N., Lin B., Shin J., Phelan P.J., Tsichlis P., Schwob J.E., Bullock P.A. (2017). The Replication of JCV DNA in the G144 Oligodendrocyte Cell Line Is Dependent Upon Akt. J. Virol..

[85] Wilczek M.P., Armstrong F.J., Mayberry C.L., King B.L., Maginnis M.S. (2021). PI3K/AKT/MTOR Signaling Pathway Is Required for JCPyV Infection in Primary Astrocytes. Cells.

[86] Lefkowitz E.J., Dempsey D.M., Hendrickson R.C., Orton R.J., Siddell S.G., Smith D.B. (2017). Virus Taxonomy: The Database of the International Committee on Taxonomy of Viruses (ICTV). Nucleic Acids Res..

[87] Moens U., Prezioso C., Pietropaolo V. (2020). Genetic Diversity of the Noncoding Control Region of the Novel Human Polyomaviruses. Viruses.

[88] Markowitz R.B., Dynan W.S. (1988). Binding of Cellular Proteins to the Regulatory Region of BK Virus DNA. J. Virol..

[89] Moens U., Johansen T., Johnsen J.I., Seternes O.M., Traavik T. (1995). Noncoding Control Region of Naturally Occurring BK Virus Variants: Sequence Comparison and Functional Analysis. Virus Genes.

[90] Olsen G., Andresen P.A., Hilmarsen H.T., Bjørang O., Scott H., Midtvedt K., Rinaldo C.H. (2006). Genetic Variability in BK Virus Regulatory Regions in Urine and Kidney Biopsies from Renal-transplant Patients. J. Med. Virol..

[91] Bethge T., Ajuh E., Hirsch H.H. (2016). Imperfect Symmetry of Sp1 and Core Promoter Sequences Regulates Early and Late Virus Gene Expression of the Bidirectional BK Polyomavirus Noncoding Control Region. J. Virol..

[92] Gosert R., Rinaldo C.H., Funk G.A., Egli A., Ramos E., Drachenberg C.B., Hirsch H.H. (2008). Polyomavirus BK with Rearranged Noncoding Control Region Emerge in Vivo in Renal Transplant Patients and Increase Viral Replication and Cytopathology. J. Exp. Med..

[93] Randhawa P., Zygmunt D., Shapiro R., Vats A., Weck K., Swalsky P., Finkelstein S. (2003). Viral Regulatory Region Sequence Variations in Kidney Tissue Obtained from Patients with BK Virus Nephropathy. Kidney Int..

[94] Bethge T., Hachemi H.A., Manzetti J., Gosert R., Schaffner W., Hirsch H.H. (2015). Sp1 Sites in the Noncoding Control Region of BK Polyomavirus Are Key Regulators of Bidirectional Viral Early and Late Gene Expression. J. Virol..

[95] Liang B., Tikhanovich I., Nasheuer H.P., Folk W.R. (2011). Stimulation of BK Virus DNA Replication by NFI Family Transcription Factors. J. Virol..

[96] Martí-Carreras J., Mineeva-Sangwo O., Topalis D., Snoeck R., Andrei G., Maes P. (2020). BKTyper: Free Online Tool for Polyoma BK Virus VP1 and NCCR Typing. Viruses.

[97] Ajuh E.T., Wu Z., Kraus E., Weissbach F.H., Bethge T., Gosert R., Fischer N., Hirsch H.H. (2018). Novel Human Polyomavirus Noncoding Control Regions Differ in Bidirectional Gene Expression According to Host Cell, Large T-Antigen Expression, and Clinically Occurring Rearrangements. J. Virol..

[98] Saitou N., Nei M. (1987). The Neighbor-Joining Method: A New Method for Reconstructing Phylogenetic Trees. Mol. Biol. Evol..

[99] Mailund T., Brodal G.S., Fagerberg R., Pedersen C.N., Phillips D. (2006). Recrafting the Neighbor-Joining Method. BMC Bioinform..

[100] Benjamini Y., Hochberg Y. (1995). Controlling the False Discovery Rate: A Practical and Powerful Approach to Multiple Testing. J. R. Stat. Soc. Ser. B Methodol..

