# Supplementary material for: Rearrangement in the Hypervariable Region of JC Polyomavirus Genomes Isolated from Patient Samples and Impact on Transcription Factor-Binding Sites and Disease Outcomes
Source: Int J Mol Sci. 2022 May 20;23(10):5699. doi: 10.3390/ijms23105699 (PMC9144386; doi:10.3390/ijms23105699)
Supplement: Supplementary file 1 [file ijms-23-05699-s001.zip › Supplemental Table S1 legend.pdf]

Supplementary Table Legend:

Table of 989 sequences and available associated data.

This table contains 989 unique JCPyV sequences, isolated from GenBank. Tissue source and patient disease classification was determined using information found in GenBank and/or in the study associated with the sequence in GenBank.

[1]Rheumatoid Arthritis; [2]JC Virus Encephalopathy; [3]Chronic Lymphocytic Leukemia; [4]Acute Lymphoblastic Leukemia; [5]Acute Myeloid Leukemia; [6]Primary Immunodeficiency Syndrome; [7]Granule Cell Neuronopathy; [8]Patient has a disease but is confirmed to not have PML; [9]JCPyV Associated Nephropathy
